# Pathophysiological Changes to the Peritoneal Membrane during PD-Related Peritonitis: The Role of Mesothelial Cells

**DOI:** 10.1155/2012/484167

**Published:** 2012-04-10

**Authors:** Susan Yung, Tak Mao Chan

**Affiliations:** Department of Medicine, University of Hong Kong, Queen Mary Hospital, Pokfulam, Hong Kong

## Abstract

The success of peritoneal dialysis (PD) is dependent on the structural and functional integrity of the peritoneal membrane. The mesothelium lines the peritoneal membrane and is the first line of defense against chemical and/or bacterial insult. Peritonitis remains a major complication of PD and is a predominant cause of technique failure, morbidity and mortality amongst PD patients. With appropriate antibiotic treatment, peritonitis resolves without further complications, but in some PD patients excessive peritoneal inflammatory responses lead to mesothelial cell exfoliation and thickening of the submesothelium, resulting in peritoneal fibrosis and sclerosis. The detrimental changes in the peritoneal membrane structure and function correlate with the number and severity of peritonitis episodes and the need for catheter removal. There is evidence that despite clinical resolution of peritonitis, increased levels of inflammatory and fibrotic mediators may persist in the peritoneal cavity, signifying persistent injury to the mesothelial cells. This review will describe the structural and functional changes that occur in the peritoneal membrane during peritonitis and how mesothelial cells contribute to these changes and respond to infection. The latter part of the review discusses the potential of mesothelial cell transplantation and genetic manipulation in the preservation of the peritoneal membrane.

## 1. Introduction

Peritoneal dialysis (PD) is an effective and affordable form of renal replacement therapy that is presently used by approximately 11% of the total global dialysis population [1]. Although PD has greatly improved the quality of life in patients with end-stage renal disease, a major disadvantage of this treatment is that PD solutions are bioincompatible and provoke peritoneal inflammation and mesothelial cell injury [2–5]. Furthermore, peritonitis is a major complication of PD and remains the single most important cause of technique failure and subsequent transfer to hemodialysis. It contributes to severe abdominal pain, hospitalization, catheter removal, and increased morbidity and mortality in PD patients. The mortality risk ascribed to PD-related peritonitis is 18% in the United States and >16% in Hong Kong [6, 7]. Peritonitis is characterized by turbidity in the dialysate effluent, abdominal pain, the presence of a white blood cell count of more than 100,000 cells/mL, of which 50% of the white blood cells are polymorphonuclear neutrophil cells, and a positive culture [8, 9]. Although the etiology of the bacteria is a determinant of morbidity and mortality in PD patients [10–12], studies have also demonstrated that peritoneal inflammation, age, residual renal function, malnutrition, and comorbidity can affect the outcome of the patient [13].

The majority of peritonitis episodes are due to a single microorganism [14, 15]. In contrast to surgical peritonitis, about half of these infections arise from Gram-positive bacteria [15], which originate from the patients' own nasopharyngeal or skin flora [16, 17]. With improvements in connection technology and better sterile techniques, Gram-positive peritonitis has gradually declined. Polymicrobial infection that involves more than one Gram-positive bacteria would suggest touch contamination or catheter infection, whereas polymicrobial Gram-negative bacteria would suggest perforation of the bowel [9, 18]. Gram-positive microorganisms that induce PD-related peritonitis include *Staphylococcus aureus* (*S. aureus*) and coagulase-negative *Staphylococcus* (CNS), that is, *S. epidermidis*, while *Pseudomonas* species, *Escherichia coli (E. coli)*, *Klebsiella* species, and *Acinetobacter* species account for the majority of Gram-negative peritonitis [19].

Peritonitis causes severe injury to mesothelial cells, specialized epithelial cells that line the peritoneal membrane and play a key role in peritoneal homeostasis, peritoneal host defense, and maintenance of the peritoneal membrane structure [2]. Compelling evidence has demonstrated that the constant exposure of the peritoneal membrane to bio-incompatible PD solutions induces peritoneal inflammation, exfoliation of mesothelial cells and structural changes to the peritoneal membrane resulting in the progressive loss of peritoneal functions and unfavorable outcome [3, 20–23]. These changes are exacerbated by peritonitis [24, 25]. We and others have demonstrated that following clinical resolution of peritonitis dialysate levels of inflammatory and fibrotic mediators remain elevated compared to preperitonitis levels [26–28], which would prolong peritoneal inflammation and mesothelial cell injury. This review will provide a brief overview of the structure and functions of mesothelial cells and how they regulate and/or contribute to peritoneal inflammation and structural changes to the peritoneal membrane during PD and peritonitis. The last section of this review will discuss the potential therapeutic interventions that may be employed to preserve the dialytic potential of the peritoneal membrane.

## 2. Peritoneal Mesothelial Cells

Although previously considered to function simply as a lubricating, nonadhesive surface to facilitate intracoelomic movement, there is now compelling evidence to show that peritoneal mesothelial cells are not inactive cells but play essential roles in peritoneal homeostasis, fluid and solute transport across the peritoneal membrane, peritoneal inflammation, and tissue repair [29–32]. Mesothelial cells synthesize a myriad of growth factors, cytokines, proteases, matrix proteins, and proteoglycans that contribute to the function of the peritoneal membrane [33–40].

Although mesothelial cells originate from the mesoderm, they possess many features of epithelial cells. Such features include the acquisition of a polygonal, cobblestone appearance, becoming polarized upon cell-cell contact and resting upon a basement membrane [29, 30]. Epithelial and mesothelial cells are also endowed with microvilli and the expression of the intermediate filament protein cytokeratin [29, 41–43]. Mesenchymal characteristics of mesothelial cells include vimentin, desmin, and *α*-smooth muscle actin expression and the acquisition of a fibroblastic phenotype following epithelial-to-mesenchymal transdifferentiation (EMT) [41, 44–46]. The ability of mesothelial cells to undergo phenotypic changes under physiological and pathological conditions underscores the plasticity property of these cells. Mesothelial cells are connected by intercellular junctions comprising tight junctions, gap junctions, adherens junctions, and desmosomes that contribute to the establishment and maintenance of a continuous mesothelial monolayer [47–49]. Reduced expression of adherens junctions during inflammatory processes is associated with a breakdown of cell-cell communication and cell-matrix interaction resulting in the denudation of the mesothelium, a process that is often observed during PD and peritonitis [50, 51]. Mesothelial cells also express E-cadherin, a cardinal feature of epithelial monolayers [52, 53]. E-cadherin is a calcium-dependent transmembrane glycoprotein localized in adherens junctions in the basolateral membrane and bestows upon mesothelial cells their apico-basolateral polarity [53, 54]. A loss of E-cadherin at the intercellular junctions is strongly associated with epithelial dedifferentiation and EMT, with the appearance of Snail, a zinc finger transcription factor that is critical for the initiation of EMT [55, 56]. Snail has been implicated in E-cadherin repression through its ability to bind to components in the promoter region of E-cadherin [57, 58]. Long-term PD and peritonitis have been shown to induce Snail expression and EMT in mesothelial cells, mediated in part through increased TGF-*β*1 bioactivation and the interaction of advanced glycation end products (AGEs) with its receptor RAGE [55, 59, 60].

The luminal surface of mesothelial cells contains numerous microvilli and occasional cilia that serve to increase the peritoneal surface area for transport of solutes across the peritoneal cavity. Microvilli entrap water and serous exudates, which protect the delicate surface of mesothelial cells from frictional damage [29]. Microvilli permit mesothelial cells to sense and respond to their microenvironment and also entrap bacteria thereby preventing infection. A reduction in the number of microvilli on mesothelial cells would therefore have a profound effect on peritoneal function and their ability to fend off bacterial infection. The density of microvilli on regenerating mesothelial cells may vary and is dependent on the anionic charge of the glycocalyx [43]. The glycocalyx is a thin film of fluid that is found on the surface of mesothelial cells. It is composed of lipoproteins, phospholipids, proteoglycans, and hyaluronan and serves to lubricate the peritoneal viscera and protect the mesothelial surface from abrasions and adhesions. The glycocalyx also plays an important role in cell-cell contact, tissue hydration, regulation of inflammation, tissue remodeling, and flow of nutrients and growth factors across the peritoneal membrane [61]. The integrity of the glycocalyx is in part attributed to the presence of negatively charged proteoglycans and hyaluronan [62, 63].

Mesothelial cells provide the first line of defense against chemical or bacterial insult to the peritoneal membrane. It is therefore crucial that following injury and denudation restoration of the mesothelium occurs promptly and is not hindered. Numerous mechanisms have been proposed for mesothelial replenishment and these include centripedal migration of mesothelial cells, exfoliation of healthy mesothelial cells from neighboring sites which settle on the denuded area, free-floating reserve cells, submesothelial and bone-marrow-derived precursor cells, and macrophage transformation [29, 64–71]. It is noteworthy that these mechanisms have been identified *in vitro* and experimental systems and their relevance in the clinical setting remain to be defined. We have demonstrated that following mechanical denudation of cultured mesothelial cells, repopulation of the monolayer is mediated through the induction of EMT in mesothelial cells at the leading edge of the wound and the migration of these cells into the denuded area [72, 73]. Increased *de novo* synthesis and subsequent deposition of hyaluronan and matrix proteins into the extracellular milieu act as a substratum that allows mesothelial cells to attach and migrate into the denuded area [72, 73]. Once the mesothelial monolayer is reestablished and cell-cell contact restored, cells resume their epithelial morphology. Although the process through which mesothelial cells revert back to their epithelial morphology has not been explored, it is likely that it is achieved by mesenchymal-to-epithelial transdifferentiation.

During peritoneal homeostasis, a fine balance exists between mesothelial injury and regeneration. Mesothelial cells are most susceptible to injury and if repopulation of the monolayer is compromised following long-term PD or recurrent episodes of peritonitis, in the absence of its protective mesothelial covering, the interstitium will initiate reparative processes that may overcompensate resulting in peritoneal fibrosis and sclerosis.

## 3. Changes to the Peritoneal Membrane during PD and Peritonitis

### 3.1. The Normal Peritoneal Membrane

The peritoneum is a delicate, continuous, and translucent membrane that lines the peritoneal cavity [74, 75]. It is composed of a monolayer of mesothelial cells resting upon a thin basement membrane, underneath which is the submesothelium comprising interwoven bundles of collagen fibres, intermittent fibroblasts, and blood vessels [74, 76]. The thickness of the submesothelium is quite variably in different sections along the peritoneum, and movement of molecules through the submesothelium is governed not only by its thickness but also by the molecular weight, charge, and shape of the molecule [76]. An in-depth discussion on the ultrastructure of the peritoneum is outside the scoop of this review and readers are referred to an excellent review by Gotloib [76].

### 3.2. Alterations in the Peritoneal Membrane during PD and Peritonitis

Many patients on long-term PD exhibit reduplication of the mesothelial and endothelial basement membranes, increased synthesis and deposition of matrix proteins within the submesothelium, and progressive subendothelial hyalinization, with narrowing or obliteration of the vascular lumen [3, 74, 75, 77]. Vascular and interstitial changes become more apparent with progressive use of PD, thereby demonstrating a temporal relationship between peritoneal fibrosis, vasculopathy, and time on PD [3]. Peritoneal fibrosis is detected in 50% and 80% of PD patients within one and two years, respectively, on PD [78–80]. With regards to mesothelial cells, independent researchers have demonstrated significant changes to these cells following their exposure to PD, which include cell activation, cell hypertrophy, increased vacuolation, partial or complete loss of microvilli, dissolution of cell-cell contacts, and alterations in the number of endoplasmic reticulum and micropinocytotic vesicles [3, 74, 75, 81]. Some degree of mesothelial denudation is invariably observed in PD patients and is associated with thickening of the submesothelium and vasculopathy [3]. What happens to mesothelial cells following their exfoliation is currently unclear. Do they represent degenerative cells that are destined for removal from the peritoneal cavity by phagocytosis or are they still viable, even though in suspension, and are able to maintain a functional role within the peritoneum? The ability to culture mesothelial cells from dialysis effluent would indicate that these detached cells are viable. The phenotypes of these cells is diverse and consist of cells with a normal epithelial morphology, large senescent cells containing multi-nuclei and multivacuoles, and cells with a fibroblastic phenotype [59].

Peritoneal specimens obtained from PD patients with peritonitis show more pronounced degenerative changes in the mesothelium and exfoliation of mesothelial cells is more prominent. In areas where mesothelial cells are still apparent, changes observed are similar to those mentioned above with a loss of microvilli and cell-cell contact [81, 82]. Peritonitis also induces the loss of the underlying basement membrane and promotes extensive interstitial fibrosis attributed to increased synthesis of matrix proteins and a concomitant loss of decorin [3, 38, 77, 83]. Acute infiltration of inflammatory cell into the submesothelium is also noted, which may account at least in part, to the expansion of the interstitial. These observations have been confirmed in animal models of experimental peritonitis [76, 84–91].

Whilst animal studies have contributed significantly to our understanding of peritoneal inflammation and injury induced by PD and peritonitis, one must also be aware of the limitations of these models. Numerous PD studies are conducted in animals that are not uremic, whereas in other studies animal models of PD-related peritonitis are conducted in animals that are not infused with PD solution. Given that peritoneal host defense mechanisms are impaired in PD patients attributed to the constant exposure of PD fluids [92], can mechanistic findings obtained in non-PD models of peritonitis be extrapolated to the clinical scenario? Even in animal models of PD-associated peritonitis, structural changes and the time that such changes occur do not replicate those observed in PD patients. In this respect, daily infusion of glucose-based PD fluid into rats for 4 weeks following an initial exposure of LPS to mimic Gram-negative peritonitis resulted in submesothelial thickening and an increase in the density and number of blood vessels [93]. However, denudation of the mesothelium and vasculopathy were not detected in this rat model of PD-related peritonitis, a finding that is often observed in PD patients with or without peritonitis [3, 81]. An excellent review by Mortier et al. summarizes the advantages and disadvantages of known experimental models of PD [94].

Vascular changes in the peritoneal membrane are commonly observed in PD patients. It has been suggested that changes in blood vessel density may directly affect the functional attributes of the peritoneal membrane. Mateijsen et al. observed an increase in blood vessel density, capillary dilation, and vessel wall thickening within the submesothelium of PD patients with peritoneal sclerosis when compared to controls [95]. Pathological alterations in the vasculature of peritoneal specimens obtained from uremic non-PD patients as well as PD patients include hyalinization of the blood vessels, vasculopathy, and submesothelial thickening [3, 77]. These abnormalities are more prominent in patients who have used PD for more than 6 years and are associated with the deterioration of peritoneal function [3, 77]. Peritoneal vascular changes in PD patients resemble alterations in the microvasculature of diabetic patients that include deposition of matrix proteins within the arterial wall and media of arterioles and reduplication of the capillary basement membrane [76, 96].

There is emerging evidence that increased synthesis of VEGF may at least in part contribute to neoangiogenesis and increased vasodilation and vessel permeability in PD patients [97, 98]. Invariably, these vascular changes are associated with the deposition of AGEs in the vessel wall, which accumulate with progressive use of PD [98]. Increased peritoneal expression and dialysate levels of VEGF are associated with increased permeability of small solutes and a loss of ultrafiltration [98–102], the increase in VEGF a result of local synthesis by peritoneal vascular endothelial and mesothelial cells [103–107]. Aroeira et al. noted that mesothelial cells isolated from dialysate effluent with a fibroblastic phenotype, (i.e., cells that have undergone EMT) synthesized significantly more VEGF than their epithelial counterpart [106]. Patients whose PD effluent contained mesothelial cells that had undergone EMT demonstrated higher serum levels of VEGF compared to levels detected in patients with mesothelial cells of normal morphology, which correlated with peritoneal transport rates [106]. The observation that these fibroblastic cells stained for cytokeratin confirms their mesothelial origin, which colocalized with VEGF expression in the submesothelium [106]. This study thus underscores the importance of mesothelial cells in the synthesis of VEGF and their contribution to vascular changes during PD. The observation that capillary tube formation in human umbilical vein endothelial cells (HUVEC) can be induced by supernatant obtained from RAGE-stimulated mesothelial cells or the coculture of RAGE-stimulated mesothelial cells with HUVEC suggests direct communication or cross-talk between mesothelial cells and endothelial cells *in vivo* and substantiates the contributing role of the former cell type in mediating neoangiogenesis and vascular changes [108].

Preliminary studies by Szeto et al. showed that VEGF levels are further increased at the onset of peritonitis in PD patients and these levels correlate with the degree of diminished ultrafiltration [109]. In an LPS-induced rat model of Gram-negative peritonitis, Pawlaczyk et al. demonstrated that the infusion of LPS at various concentrations together with PD solution increased dialysis effluent concentrations of VEGF in a dose-dependent manner [110] but its role in mediating changes in the peritoneal vasculature was not investigated.

The causal relationship between peritonitis and functional changes of the peritoneal membrane is controversial. Whilst some studies have demonstrated a causal relationship between peritonitis rate and peritoneal dysfunction, others have failed to find any correlation [24, 111–115]. These discrepancies may be related to the limited patient number, short period of followup, insufficient longitudinal studies, and grouping Gram-positive and Gram-negative complicating peritonitis together. Ates et al. assessed the degree of peritoneal function remaining in 18 PD patients over the course of 24 weeks following onset of infection. These researchers demonstrated that one episode of peritonitis was insufficient to induce permanent changes in peritoneal transport properties although full recovery of ultrafiltration was not achieved following the resolution of peritonitis [116]. In another study, data obtained from Davies et al. confirmed that one episode of peritonitis is not sufficient to have any significant effect on peritoneal function, whereas recurrent peritonitis that occurred in close proximity and the severity of peritoneal inflammation exacerbated and accelerated solute transport and the loss of ultrafiltration in PD patients [24]. It is noteworthy that since the structural and functional properties of the peritoneal membrane vary significantly between patients, their response to peritonitis, even towards the same pathogen, can differ considerably.

### 3.3. Role of Anionic Sites in the Peritoneal Membrane during PD and Peritonitis

Accumulation of plasma proteins in the peritoneal cavity and their subsequent loss from the patient following the exchange of PD fluid is often observed during peritonitis [85, 117]. It can perhaps be likened to proteinuria whereby a loss of heparan sulfate proteoglycans, such as perlecan or agrin, in the glomerular basement membrane (GBM) results in the increased permeability of the GBM to anionic macromolecules such as albumin [118, 119]. Proteoglycans are anionic macromolecules that comprise a core protein to which one or more glycosaminoglycan chain(s) is/are attached [120]. Glycosaminoglycan chains are classified as heparan sulfate, chondroitin sulfate, dermatan sulfate, keratan sulfate, and hyaluronan depending on their disaccharide units. With the exception of hyaluronan, all glycosaminoglycan chains are attached to a protein core and are endowed with sulfate groups that bestow up these macromolecules a high net negative charge that contributes to their biological properties and interactions with cytokines, chemokines, and growth factors [121, 122]. Perlecan and agrin are the most characterized heparan sulfate proteoglycans in the GBM, which contribute to the structural integrity of the kidney and restrict the passage of albumin and other proteins out of the glomerular capillaries into the urinary space [118, 119]. Through their ability to sequester chemokines, heparan sulfate proteoglycans can also regulate lymphocyte recruitment during tissue injury [123]. Therefore, changes in the expression of proteoglycans in any given tissue will have a profound effect on both their structural and functional property.

Gotloib et al. provided evidence that anionic sites exist in the normal peritoneum and are found within the mesothelial glycocalyx, subendothelium, and along the basement membrane [124–126]. In an experimental model of septic peritonitis whereby rats were administered live *E. coli* by intraperitoneal injection, a significant reduction in anionic sites at these locations was observed, which was accompanied by increased transperitoneal passage of proteins [85]. Although the nature of these anionic sites was not further investigated by these researchers, it is possible that perlecan may contribute at least in part to the anionic staining. We have demonstrated that perlecan expression is predominately observed within the mesothelium and underlying basement membrane in peritoneal specimens obtained from new PD patients [38]. Mesothelial expression of perlecan decreased with increasing duration on PD with a concomitant increase in the submesothelium [38]. The functional role of perlecan in the mesothelium remains to be fully elucidated, but it is possible that perlecan plays a critical role in preserving the structural and functional integrity of the peritoneum and maintenance of the selective charge barrier of the peritoneal membrane. Emerging evidence suggests that heparan sulfate proteoglycans may possess angiogenic properties [122, 127]. Through their N-terminal, heparan sulfate proteoglycans may stimulate angiogenesis through their ability to bind growth factors such as VEGF, bFGF, and PDGF and presenting them in a biologically active form to their cognate receptors [127]. With regards to the observed increase in perlecan expression in the submesothelium in PD patients, it is plausible to suggest that it may play a role in peritoneal angiogenesis although further studies are warranted to confirm this. Whether the expression of perlecan in the submesothelium is further increased following peritonitis remains to be determined.

## 4. Peritoneal Inflammation

Inflammation is the body's adaptive response to remove the inciting insult and restore homeostasis to the tissue. Given the molecular heterogeneity of bacteria pathogens, it is quite remarkable that efficient and coordinated recognition strategies have evolved to deal with bacterial infection, and this is based primarily on the ability of the host to detect molecular patterns that are unique to bacteria. If the epithelial barrier is breached, the pathogen is eliminated by the innate immunity, followed by the adaptive host immune processes. Innate immunity consists of various preexisting, rapidly mobilized cells that include the immune cells, that is, neutrophils, macrophages, mast cells, eosinophils and natural killer cells, and resident cells. These cells express a number of pattern recognition receptors such as the toll-like receptors (TLRs) that are activated by microbial components resulting in complement activation and the release of proinflammatory mediators such as cytokines, chemokines, nitric oxide, prostaglandin, acute phase proteins, and antimicrobial peptides. Cells of the innate immune system subsequently activate the adaptive immune system, which initiate the maturation of dendritic cells and recruitment of T and B cells [128]. Under normal circumstances, cell infiltration is initiated within minutes of detecting a bacterial insult and through a coordinated series of cellular and humoral events [129], the inciting insult is removed with a systematic return of the normal physiological functions of the tissue within days. With successful resolution of inflammatory processes, the extent of tissue damage is limited. If the inciting insult persists, chronic inflammation ensues, which may persist for weeks, months, or even years.

### 4.1. The Role of Infiltrating Cells in Peritoneal Inflammation

Before the processes of bacterial-induced peritoneal inflammation and the involvement of infiltrating and mesothelial cells in these processes are discussed, one must remember that the peritoneal host defense system of PD patients is already compromised. *In vitro* and *ex vivo* studies have demonstrated that PD fluids have a marked effect on macrophage functions. In this respect, PD fluid has been shown to induce cytokine secretion and inhibit the respiratory burst and phagocytotic property of peritoneal macrophages [130, 131]. Furthermore, McGregor et al. demonstrated that peritoneal macrophages became increasing immature with increasing time on PD, which was accompanied by an increase in cytokine release [130]. Components of PD solution have also been shown to alter leukocyte functions and are cytotoxic to these immune cells [132, 133]. Daily use of PD not only dilutes the number of macrophages and leukocytes and concentration of opsonins in the peritoneal cavity of PD patients, but these cells and components are lost from the peritoneal cavity after each PD exchange, further compromising peritoneal host defense.

How does onset of infection affect the inflammatory processes and the structural and functional properties of the peritoneum when on-going peritoneal inflammation is already present? Is it possible to dissect out subtle changes that occur within the peritoneum consequent to peritonitis from those that have already arisen from the use of long-term PD? Whilst it is impractical to obtain frequent peritoneal biopsies from PD patients to monitor the morphological changes within the peritoneal membrane with time on PD and during episodes of infection, we rely on the measurement of surrogate markers in dialysate effluent that may provide limited insight in the inflammatory processes that occur within the peritoneum. The use of transgenic mouse studies has provided us with intricate details of the roles of inflammatory and fibrotic mediators and their ligands synthesized by immune and resident cells, in inflammatory and reparative processes of the peritoneum [134–136].

The initial recognition of an infection in the peritoneum is mediated by peritoneal macrophages and mast cells, triggered by TLR activation, which results in the release of various vasoactive substances such as prostaglandins and histamines. This results in vasodilatation and increased permeability of the peritoneal blood vessels leading to increased synthesis of complement, immunoglobulins, opsins, fibrin, and clotting factors [25]. Peritoneal macrophages also synthesize various inflammatory mediators that include IL-1*β*, TNF-*α*, IFN-*γ*, and TGF-*β*1, which in turn mediate the induction of chemokine secretion. The main effect of these mediators is to elicit the recruitment of polymorphonuclear neutrophils that are normally restricted to the blood vessels to the site of injury [129]. These neutrophils are activated either by direct contact with the invading pathogen or through the actions of the cytokines and chemokines secreted by resident mesothelial cells. In order to eradicate the pathogen, neutrophils release the toxic content of their granules which include reactive oxygen and nitrogen species, cathepsin G, and elastase [137]. These potent effectors do not discriminate between pathogens and host cells and therefore injury to the peritoneal membrane is unavoidable. Neutrophils are subsequently progressively cleared from the peritoneal cavity by apoptosis and are replaced by a population of monocytes/macrophages and leukocytes. Ingestion of neutrophils by macrophages results in the release of TGF-*β*1, its mode of action now becoming anti-inflammatory where it assists in the reparative processes [138]. Repopulation of resident macrophages in the peritoneal cavity following the resolution of peritoneal inflammation has recently been shown to be through local proliferation [139].

The temporal switch in immune cell population is pivotal for the clearance of infection and resolution of inflammation, mediated in part through IL-6, a cytokine known to prevent the accumulation of neutrophils. Hurst et al. demonstrated that leukocyte recruitment into the peritoneal cavity is mediated by the interaction of soluble IL-6R and IL-6, shed from infiltrating neutrophils and mesothelial cells, respectively, which in turn induce chemokine expression essential for lymphocyte recruitment [140]. Over the last decade or so, our understanding of the mechanisms through which neutrophils are recruited from the circulation, migrate across the submesothelium into the peritoneal cavity, and the sequential change in the population of infiltrating cells to initiate peritoneal inflammation and resolution has increased significantly through a series of in-depth *in vitro, ex vivo,* and experimental studies [134–136, 141–144].

### 4.2. The Role of Mesothelial Cells in Peritoneal Inflammation

Both infiltrating and resident peritoneal cells play critical roles in the initiation and amplification of peritoneal inflammation during PD and peritonitis through their ability to synthesize chemotactic and proinflammatory mediators [4, 33–35]. Whilst the roles of infiltrating cells in peritoneal inflammation have been described above, the next section will focus on the resident mesothelial cells and their contribution to cellular infiltration and initiation and resolution of peritoneal inflammation.

The initiation phase is characterized by the activation of resident macrophages and mesothelial cells by the invading microorganism or its secreted products. The initiation phase is followed by the amplification phase, in which mesothelial cells play a predominant role. Mesothelial cells are activated by proinflammatory cytokines, such as TNF-*α* and IL-1*β* derived from peritoneal macrophages [25]. Stimulation of mesothelial cells by TNF-*α* or IL-1*β* induces IL-8 production, a potent chemoattractant that mediates leukocyte migration from the circulation to the peritoneal cavity [145, 146]. Mesothelial cells constitutively express adhesion molecules, such as ICAM-1, VCAM, and PECAM, which are required for leukocyte adherence and migration across the mesothelium [147–149], a multistep process that is dependent on the establishment of a chemotactic gradient across the mesothelium, increased mesothelial expression of ICAM-1, and specific adhesive interactions between the leukocytes and the endothelium [146, 150].

CD40 is a member of the TNF family of receptors and is expressed on the membrane of activated CD4^+^ T-cells. Its activation contributes to increased chemokine and cytokine secretion during inflammatory processes and binding to its ligand initiates mononuclear cell infiltration during peritonitis [143]. Basok et al. demonstrated that mesothelial cells also express CD40 [151]. Activation of CD40 on mesothelial cells by proinflammatory cytokines induced IL-15 secretion, a T-cell growth factor and activator [152]. The presence of CD40 on mesothelial cells may suggest that these cells play an important role in T-cell regulated inflammatory response during peritonitis.

In the previous section, the importance of macrophages in the initiation and resolution of peritonitis was highlighted. Once the pathogen is eliminated, resolution of peritoneal inflammation may begin. Rapid and effective clearance of macrophages dictates the duration of peritoneal inflammation and may be an important determinant of chronic peritoneal inflammation. Unlike neutrophils which are removed by apoptosis, macrophage clearance is through emigration into the draining lymphatics [153]. Recently, Bellingan et al. demonstrated in a murine model of peritonitis that macrophages adhered specifically to the peritoneal mesothelium through VLA-4 and VLA-5 and this interaction was RGD sensitive [154]. The adhesion of macrophages to the mesothelium was localized to areas overlying the draining lymphatics, was adhesion molecule dependent, and the rate of emigration was controlled by the level of macrophage activation [154]. Mesothelial-macrophage interactions are therefore prerequisite for the removal of macrophages from the peritoneal cavity and for the resolution of peritoneal inflammation.

We and others have demonstrated that increased levels of cytokines and growth factors may persist in the peritoneal cavity despite clinical resolution of peritonitis [26–28], which will prolong injury to the mesothelial cells. This will initiate the fibrogenic phase within the peritoneum, which is followed by the destruction phase. Overproduction of matrix proteins in the peritoneum will result in peritoneal fibrosis and sclerosis and invariably lead to cessation of treatment.  Table 1 summarizes the detrimental processes that occur during each phase.

## 5. The Role of Defensins during Peritonitis and Peritoneal Inflammation

Numerous host proteins have been shown to possess antimicrobial activity. Many are constitutively expressed by resident cells and stored in secretory granules, whereas others are induced upon proinflammatory stimuli. Defensins are a group of antimicrobial peptides that are produced by mesothelial cells and cells of the innate immune system in response to bacterial infection [155, 156]. These peptides are activated in the presence of bacteria and act by disrupting the lipid membranes of bacteria. In order to avoid elimination from the host, bacteria have developed mechanisms that allow them to utilize components of the host cells to enhance their virulence. In this respect, pathogens have been shown to exploit cell surface proteoglycans, which have a high net negative charge, to neutralize the antimicrobial actions of cationic defensins. Schmidtchen et al. demonstrated that exogenous dermatan sulfate and heparan sulfate glycosaminoglycans are able to bind *α*-defensin, which fully neutralized its bactericidal activity against *P. aeruginosa, E. faecalis,* and *S. pyogenes* [157]. These researchers further demonstrated that through the actions of their proteinases, these pathogens have the capacity to utilize and degrade host-derived proteoglycans to release anionic glycosaminoglycan chains that bind and neutralize the actions of defensins [157]. Syndecan-1 is a cell surface heparan sulfate proteoglycan that is synthesized by fibroblasts, mesothelial cells, airway epithelial cells, and intestinal epithelial cells [158–160]. In an animal model of *S. aureus* corneal infection, Hayashida et al. noted that *S. aureus* induced shedding of the syndecan-1 ectodomain, which resulted in the inhibition of the innate immune mechanism and the inability of neutrophils to eradicate *S. aureus* [161]. The observation that syndecan-1 knockout mice were resistant to *S. aureus* infection underscores the importance of syndecan-1 shedding as a pathogenic mechanism that mediates virulence of *S. aureus* [161]. *P. aeruginosa* is also able to release the ectodomain of syndecan-1 from the cell surface of mouse mammary epithelial cells, lung epithelial cells, and fibroblasts using LasA, a zinc metalloendopeptidase [162], and this has been implicated as a pathogenic mechanism that permits *P. aeruginosa* to mediate tissue injury in the lung and cornea. Given that *S. aureus* and *P. aeruginosa* are common pathogens that induce peritonitis in PD patients, it is possible that both microorganisms implement the shedding of syndecan-1 ectodomain from the mesothelium to promote their pathogenesis during peritonitis. In this regard, our preliminary studies have demonstrated increased levels of glycosaminoglycans in dialysis effluent obtained from patients with peritonitis. It is also plausible to suggest that this mechanism may also account in part for the loss of anionic sites from the mesothelium in experimental models of peritonitis. 

Mesothelial cells are a major contributor of defensin production in the peritoneum but their antimicrobial function role in the peritoneum remains to be fully elucidated. Denudation of mesothelial cells from the peritoneal membrane during PD and peritonitis would suggest a concomitant loss of defensin production, but thus far, this does not appear to have any impact on the incidence of peritonitis [163]. In addition to their antimicrobial activity, defensins are thought to possess chemoattractant properties for immature dendritic cells and indirectly contribute to leukocyte infiltration by activating resident cells to secrete proinflammatory chemokines and cytokines [164–166]. It is therefore possible that defensins may contribute to both the innate and adaptive immune responses in the peritoneum although further studies are warranted to confirm this.

## 6. Effect of Peritonitis on Mesothelial Cells

There is compelling evidence to show that mesothelial cells play an essential role in the orchestration of peritoneal responses during inflammation and peritonitis. Changes to the structural and functional integrity of the mesothelium during PD and infection will therefore have a profound effect on how peritonitis is resolved. In order to delineate the mechanisms through which mesothelial cells regulate peritoneal inflammation and infection, and the underlying mechanisms through which peritonitis can modulate the structural and functional integrity of the mesothelium, the establishment of a reproducible model that can mimic the *in vivo* environment is essential to allow researchers to perform experiments in a controlled manner. Mesothelial cells isolated from omental specimens possess identical biochemical and morphological characteristics to those identified in peritoneal mesothelial stem cells. Therefore, cultured mesothelial cells provide a relevant tool to study the underlying mechanisms through which pathogens alter the structural and functional properties of the mesothelium. It is noteworthy that mesothelial cells isolated from mature donors have an inflammatory phenotype even in the absence of any stimuli [167] and therefore it is imperative when assessing inflammatory processes that one can distinguish between changes induced by the inciting stimulus and that by the age of the cells.

Previous studies have shown that different causative microorganisms of peritonitis are associated with distinct clinical outcomes and therefore should not be considered comparable in terms of outcome [10]. Troidle et al. reported that patients with Gram-positive peritonitis fared better on PD compared to patients with Gram-negative peritonitis since the latter was associated with a greater need for hospitalization and catheter removal, and higher incidences of relapse and mortality [10]. It is therefore conceivable that Gram-positive and Gram-negative bacteria will have distinct effects on the mesothelium and therefore one should not ideally collate data from Gram-positive and Gram-negative bacteria together.

*γδ* T-cells constitute approximately 0.5–5% of the total human peripheral blood T-cell population and are activated by small nonpeptide phosphoantigens such as (E)-4-hydroxy-3-methyl-but-2-enyl pyrophosphate (HMB-PP) [168]. HMB-PP is produced predominantly in Gram-negative bacteria [169]. HMB-PP-dependent cross-talk between V*γ*9/Vv2 T-cells and autologous monocytes has recently been shown to drive the induction of chemokine and cytokine secretion and induce the differentiation of monocytes to inflammatory dendritic cells [169]. It is thus possible that HMB-PP may contribute to the observed difference between the severity of peritoneal inflammation in Gram-positive and Gram-negative bacteria although further studies are warranted to confirm this.

### 6.1. Toll-Like Receptors and Nucleotide-Binding Oligomerization Domain- (Nod-) Like Receptors

TLR play important roles in the initial recognition of bacterial, viral, and fungal components in the host defense system, and ten TLRs have been identified thus far. Independent researchers have demonstrated that both human and murine mesothelial cells constitutively express mRNA for TLR1-6, whilst TLR7-10 are barely detectable [170, 171]. TLR4 recognizes LPS, a major component of the outer membrane of Gram-negative bacteria. In an attempt to delineate the biological role of TLR4 during infection, Kato et al. administered LPS to C3H/HeN and C3H/HeJ mice by intraperitoneal injection, the latter strain being hyposensitive to LPS due to a point mutation in the TLR4 gene and investigated its effect on inflammatory processes. The observation that NF*κ*B activation, induction of MCP-1 and MIP-2 secretion, and recruitment of leukocytes into the peritoneal cavity was dependent on TLR4 highlights its importance in peritoneal inflammation [170]. Recently, Colmont et al. demonstrated that human peritoneal mesothelial cells are able to respond to Gram-positive and Gram-negative bacterial ligands through TLR2 and TLR5, respectively [171]. Unlike murine mesothelial cells, human mesothelial cells demonstrated a lack of TLR4 responsiveness to LPS [171]. Although the functional consequences of these interactions remain to be determined, given that Gram-positive and Gram-negative bacteria induce different clinical outcomes in PD patients, is it possible that their recognition by mesothelial cells through distinct TLR subsets may induce distinct inflammatory processes within the peritoneum and which may explain, at least in part, the observed differences in clinical outcome?

Sensing of bacterial pathogens by mesothelial cells may also be mediated by nucleotide-binding oligomerization domain- (Nod-) like receptors [172]. Whilst TLR mediates the recognition of bacterial components either at the cell surface or in endosomes, Nod-like receptors induce the innate immune system through cytosolic recognition of bacterial constituents [173]. Recently, in transgenic animal studies, Park et al. demonstrated that Nod-1 and Nod-2 can regulate chemokine and antibacterial innate immune responses in mesothelial cells through the kinase RICK/RIP2 pathway, which mediated the downstream activation of NF-*κ*B and MAPK pathways [172]. The ability of mesothelial cells to respond to bacterial components to initiate inflammatory responses highlights their pivotal role in peritoneal host defense.

### 6.2. Induction of Inflammatory and Fibrotic Mediators

Peritonitis has been shown to induce local production of various inflammatory and fibrotic mediators in mesothelial cells. Although the list of mediators is ever increasing, we will focus on some mediators that have been suggested to possess dual roles in inflammation and fibrosis. Hyaluronan is a negatively charged, linear polysaccharide that is widely distributed in epithelial, connective, and neural tissues [174, 175]. In normal tissues, hyaluronan is synthesized as a macromolecule with a molecular weight in excess of 10^6^ Da [176]. Despite its simple structure, hyaluronan is a multifaceted molecule that contributes to the structural integrity of tissues, maintains water balance, and possesses anti-inflammatory properties [177, 178]. High-molecular-weight hyaluronan undergoes steady-state turnover and its degradation into small, nonbiologically active fragments is rapidly removed from the body by the liver. In chronic inflammation, elevated serum levels of hyaluronan and its deposition at sites of injury have been observed. Fragmentation of extracellular matrix (ECM) components often occurs during tissue injury and these fragments possess functional properties that are distinct from their parent molecule [178]. Removal of ECM fragments from the tissue is therefore vital for the resolution of tissue injury. Independent researchers have suggested that hyaluronan fragments may deposit in inflamed tissues consequent to their *de novo* synthesis or through the depolymerization of native hyaluronan following increased activity of hyaluronidase or reactive oxygen species [178–180]. Unlike native hyaluronan, hyaluronan fragments have been shown to induce multiple signaling cascades and increase cell proliferation, cytokine secretion, MMP activity, and matrix protein synthesis in murine models of lung disease or cultured mesothelial cells, keratinocytes, macrophages, and dendritic cells [178, 181–187]. Proinflammatory cytokines and profibrotic growth factors have been shown to increase synthesis of both high- and low-molecular-weight hyaluronan in various cell types [188–191]. Studies have also demonstrated that TLR-2, TLR-4 and nod-like receptors can detect low molecular weight hyaluronan, and through these interactions are able to initiate inflammatory responses in an animal model of lung injury, whereas over-expression of high molecular weight hyaluronan was shown to maintain epithelial cell integrity and promote recovery [192].

Hyaluronan is a surrogate marker of inflammation. We and others have demonstrated that low levels of hyaluronan can be detected in dialysis effluent obtained from noninfected PD patients, and these levels are significantly increased during peritonitis [193, 194]. Our observation that dialysate hyaluronan levels are almost 2- and 10-folds higher that the corresponding serum levels in noninfected and infected PD patients, respectively, suggests that hyaluronan is synthesized locally [193]. We have demonstrated that cultured mesothelial cells can synthesize hyaluronan in abundance, of which 90% is secreted into their culture medium. The observation that the hydrodynamic size of hyaluronan synthesized by mesothelial cells is identical to that of hyaluronan detected in PD fluid, together with our observation that infected PD fluids can induce *de novo* synthesis of hyaluronan in these cells, provides evidence that mesothelial cells contribute to the increased local synthesis of hyaluronan during PD and peritonitis [193]. The molecular weight of hyaluronan present on the surface of mesothelial cells is higher than the secreted form, suggesting partial depolymerization of the parent molecule as it is released from the plasma membrane. Once released, hyaluronan appears stable and does not undergo further fragmentation in the peritoneal cavity during chronic PD or peritonitis [193]. Increased synthesis of hyaluronan in mesothelial cell during peritonitis is attributed to their induction by proinflammatory cytokines and growth factors, in particular, IL-1*β* IL-6, TNF-*α*, TGF-*β*1, and PDGF [188, 194]. The inability to detect hyaluronan fragments in spent infected and noninfected dialysate corroborates previous reports that low-molecular-weight hyaluronan is rarely observed in injured tissue *in vivo* [195]. It is noteworthy that studies detailing hyaluronan fragments as inflammatory mediators predominantly stem from *in vitro* studies [178, 183, 184, 196–199] and thus their existence in injured tissues and clinical relevance remains to be defined.

We have demonstrated that increased hyaluronan levels can induce EMT in mesothelial cells under physiological conditions, which is essential for cell migration during wound healing and remesothelialization [73]. Once the mesothelial monolayer is restored, hyaluronan levels are reduced [73]. A sustained increase in hyaluronan levels within the peritoneum during peritonitis would imply prolonged activation of mesothelial cells thereby preventing mesenchymal-to-epithelial transdifferentiation and their ability to revert back to their epithelial morphology. The acquisition of a migratory and invasive phenotype allows mesothelial cells to adopt a more fibrogenic characteristic whereby synthesis of MMPs that degrade the underlying basement membrane is increased, thus permitting the migration of transdifferentiated mesothelial cells into the submesothelium [59]. In this respect, Fukudome et al. demonstrated increased MMP-9 activity in dialysis effluent obtained from PD patients with peritonitis [200]. Transdifferentiated mesothelial cells have been shown to contribute to the thickening of the submesothelium and subsequent peritoneal fibrosis [55, 201]. Their migration into the submesothelium may also contribute to the denudation of the mesothelium, although this warrants further investigation.

Apart from hyaluronan, mesothelial cells also synthesize and secrete TGF-*β*1, IL-1*β*, IL-6, and TNF-*α* [26, 33–35, 55, 202] and their levels are increased during peritonitis. These peptides have also been shown to induce EMT in mesothelial cells and further augment peritoneal inflammation and fibrosis [55, 84, 93, 203–205]. Failure to restore the mesothelial monolayer is associated with unfavorable structural and functional changes to the peritoneal membrane of PD patients [3]. The role of TGF-*β*1 in the pathogenesis of peritoneal fibrosis is well documented [55, 205]. TGF-*β*1 also possesses an anti-inflammatory property but its ability to regulate peritoneal inflammation has been less characterized. In an animal model of peritonitis induced by *E. Coli*, Wang et al. noted a transient increase in TGF-*β*1 in the peritoneum that was associated with the activation of TGF-*β*1 and NF*κ*B signaling pathways, increased secretion of TNF-*α*, and impaired peritoneal function [206]. Resolution of peritonitis was observed after 7 days without progressing to peritoneal fibrosis. In rats whereby TGF-*β*1 signaling pathways were block by genetic manipulation, *E. coli*-induced peritonitis exacerbated peritoneal inflammation as demonstrated by increased infiltration of leukocytes and further induction of inflammatory signaling pathways and secretion of TNF-*α* [206]. These data would suggest that TGF-*β*1 may also exert a protective, anti-inflammatory activity on the peritoneum during peritonitis. The role of TGF-*β*1 in immune tolerance and in particular in the inhibition of T-cell mediated immunopathology was first demonstrated over 2 decades ago in TGF-*β*1, deficient mice, which developed an early and fatal multifocal inflammatory disease [207, 208]. TGF-*β*1 can induce T-reg cell differentiation, but in the presence of IL-6, TGF-*β*1 induction of T-reg cells is inhibited [209]. TGF-*β*1 together with IL-6 has been shown to induce Th17 cells, a subset of T helper cells that have been implicated in autoimmune disease [209, 210]. Th17 cells have also been shown to synthesis IL-17 A, IL-17F, and IL-22 following infection, and these cytokines are involved in the recruitment and activation of neutrophils and tissue homeostasis [211]. The mechanisms that dictate whether TGF-*β*1 should follow an anti-inflammatory or profibrotic pathway remain to be determined.

HGF is a growth factor that has antifibrotic and profibrotic properties depending on the cell type. It has been shown to attenuate renal fibrosis by suppressing the actions of TGF-*β*1, slow the progression of diabetic nephropathy in db/db mice, and ameliorate podocyte injury and proteinuria in a murine model of chronic progressive glomerular disease [212–215]. On the other end of the spectrum, HGF has also been shown to induce cell proliferation and EMT in endothelial cells and hepatocytes [216, 217]. Rampino et al. demonstrated that HGF induced cell proliferation, EMT, and collagen synthesis in mesothelial cells, thus indicating a pro-fibrotic role for HGF in the mesothelium. These researchers also observed an increase in the levels of HGF in dialysis effluent obtained from patients with peritonitis when compared to levels detected in non-infected PD fluid. This would indicate that HGF may contribute to the denudation of the mesothelium and increase fibrogenesis during peritonitis [218].

Angiotensin II is a potent vasoactive peptide that plays a critical role during renal fibrosis and peritoneal injury [219, 220]. Its levels are increased during peritonitis and angiotensin II has been shown to induce ERK1/2 and p38 MAPK activation and fibronectin synthesis in mesothelial cells, thereby contributing to peritoneal inflammation and fibrosis, respectively [221].

### 6.3. Alterations in the Fibrinolytic Cascade

Mesothelial cells play a critical role in maintaining the balance between fibrin accumulation and degradation through the expression of plasminogen activators, namely, tissue-type plaminogen activator (tPA) and urokinase plaminogen activator (uPA), and their specific inhibitor plasminogen activator inhibitor type 1 (PAI-1) [222]. Depending on the fibrinolytic capacity of mesothelial cells during peritonitis, fibrin may be lysed, which promotes healing, or deposited within the peritoneal structure where they induce fibroblast proliferation and collagen deposition, which inevitably results in peritoneal fibrosis [223, 224]. Most often that not, during peritoneal inflammation and peritonitis, a peritoneum devoid of its mesothelium and therefore devoid of its fibrinolytic mechanism is accompanied by the accumulation of fibrin within the peritoneum. If it is not removed, the fibrin will be replaced by granulation tissue, which in turn will be substituted by dense fibrous matter [225]. Studies have demonstrated that *S. aureus* complicating peritonitis can contribute to peritoneal fibrosis by their ability to produce a self-protecting coagulase that initiates the clotting of plasma and generation of a thrombin-like substance that permits the conversion of fibrinogen to fibrin [223, 225]. *In vitro* studies have shown that upon stimulation with proinflammatory mediators, such as IL-1*α* and TNF-*α*, tPA synthesis is inhibited in mesothelial cells, which is accompanied by an increase in PAI-1 synthesis [226, 227]. Furthermore, TGF-*β*1 has been shown to increase gene and protein expression of PAI-1 in cultured mesothelial cells, which enhanced fibrin deposition [222]. These cytokines are all increased in the peritoneum during peritoneal inflammation and peritonitis and may thus impede fibrin degradation *in vivo*. Recently, Haslinger et al. provided evidence that simvastatin could abrogate the suppressive effect of TNF-*α* on tPA synthesis in cultured mesothelial cells [228], but in the clinical setting, it is unlikely to be of much benefit if the mesothelium is already denudated.

### 6.4. Injury to Mesothelial Cells

The mesothelium plays an essential role in peritoneal homeostasis and host defense against infection. Prolonged use of PD and recurrent episodes of peritonitis result in the denudation of the mesothelium [3, 229]. In contrast to noninfected PD, where exfoliated mesothelial cells still remain viable in the peritoneal cavity, and which may have the potential to reestablish the mesothelium, emerging evidence has shown that invading pathogens such as *S. aureus* can induce caspase-independent mesothelial cell death [230]. Haslinger-Löffler et al. investigated the ability of various laboratory strains of *S. aureus* and *S. epidermidis* to induce mesothelial cell death. These researchers demonstrated that only *S. aureus* with an invasive and hemolytic phenotype induced mesothelial cell death, whereas none of the strains of *S. epidermidis *demonstrated any cytotoxic effect on mesothelial cells [230]. *S. aureus* has developed a number of mechanisms that allow the pathogen to adhere to the host cell, which is essential for progression of infection. Adhesion of *S. aureus* to components of the host cell is mediated by adhesins [231]. Fibronectin has been shown to mediate *S. aureus* attachment and subsequent invasion in mesothelial cells, an observation also noted in endothelial cells [231, 232]. In an intact, polarized mesothelium, fibronectin is normally localized to the basolateral cell membrane and therefore not readily available from the luminal aspect of the cell. In the context of PD and peritonitis, it is plausible to suggest that due to a compromised mesothelium with reduced synthesis of tight junctions, extracellular fibronectin may be exposed and accessible for *S. aureus* to bind, although this warrants further investigation. We have previously demonstrated that fibronectin is also present on the surface of mesothelial cells [40], which may also contribute to the binding and invasion of *S. aureus*. The molecular mechanism of *S. aureus* invasion bears remarkable similarities to complement-enhanced phagocytosis mediated by *β*_2_-integrins in macrophages and neutrophil granulocytes [231]. The pathogen is engulfed by pseudopodia in a time-, dose-, and temperature-dependent manner [233] and can be located in vacuoles within mesothelial cells without being digested. This is dependent on the strain of bacteria, for example, *S. aureus* Cowan I can remain in the host cell without inducing any visible signs of cell injury, whilst other strains (*S. aureus* ST 239) can induce cell and nuclear shrinkage, vacuolization, and chromatin condensation [230]. Studies have demonstrated that at low concentrations of bacteria, cell death may be mediated by caspase activation and apoptosis, whereas higher concentrations result in necrotic cell death [234]. Haslinger-Löffler et al. demonstrated that *S. aureus* with a hemolytic phenotype mediated mesothelial cell death through necrosis [230]. Increased levels of TNF-*α* and Fas ligand during peritoneal inflammation and peritonitis may also induce apoptosis in mesothelial cells [235]. In our preliminary studies, we have demonstrated that Gram-negative bacteria such as *P. aeruginosa, K. pneumoniae *and* E. coli* induced denudation of the mesothelial monolayer and cell lysis more prominently that Gram-positive species (Yung and Chan, unpublished data). If cell death through apoptosis or necrosis supersedes cell proliferation, the replenishment of mesothelial cells will be insufficient, which will initiate subsequent peritoneal fibrosis.

## 7. Potential Role of Mesothelial Cell Transplantation and Gene Therapy in Peritoneal Preservation

It is without doubt that mesothelial cells play a crucial role in numerous cell processes in the peritoneum and a loss of mesothelial cells is accompanied by impairment of the structural and functional integrity of the peritoneal membrane. The omentum is a highly vascularized tissue that has been used in reconstructive surgery over the past two decades [236–238]. How the omentum facilitates the healing process remains to be fully defined, but it has been suggested that mesothelial cells may secrete growth factors at sites of injury or are themselves incorporated into the tissue. In order to preserve the dialytic efficacy of the peritoneum during PD and peritonitis, due to encouraging results obtained with the use of the omentum in reconstructive surgery, it is perhaps possible to transplant cultured mesothelial cells into the peritoneum once the structure of the peritoneal membrane is compromised. Independent researchers have suggested that genetic engineering may offer a novel therapeutic strategy, whereby omental specimens from predialysis patients are removed at the time of catheter implantation and mesothelial cells isolated and stored frozen until required [93, 239]. *Ex vivo *gene therapy may potentially bestow upon the peritoneal membrane an increased healing property or replenish proteins crucial for the maintenance of the mesothelium, which are lost during PD. Following peritonitis episodes when denudation of the mesothelium is prominently observed, genetically modified mesothelial cells may be infused into the peritoneal cavity through the catheter, allowed to settle on the denuded tissue and repopulate the peritoneal membrane. This stimulating concept is timely since it is currently impossible to completely dialyzed patients with PD solutions free of glucose and mesothelial injury will inevitable always emerge. In reality, is such a technique feasible in PD patients? Although mesothelial transplantation in an animal model of peritonitis has provided us with some encouraging results, it was also accompanied by deranged changes to the structure of the peritoneum with induction of inflammatory processes and activation of the peritoneum [240], which rather alarmingly this technique was specially intended to prevent. Before clinical trials can even be considered, it is essential that we determine the mechanisms by which transplanted mesothelial cells are activated in order to devise approaches to inhibit such activation.

In an in-depth morphologic study of the peritoneal membrane, Williams et al. observed that of the peritoneal biopsies analyzed, mesothelial denudation was noted in 18.1% of specimens obtained from predialysis and hemodialysis patients, and in specimens that presented with an intact mesothelium, the cells assumed a reactive state [3, 229]. Although the mechanisms that results in mesothelial denudation in predialysis and haemodialysis patients have yet to be fully identified, given that chronic inflammation is a common feature of patients with end-stage renal failure [241–245], it is possible that local ischemia, uremia, and systemic inflammation may result in increased levels of proinflammatory mediators and growth factors within the peritoneum, possibly derived from the circulation or local production, which induce cell detachment. These mediators include TNF-*α*, IL-1*β*, and TGF-*β*1, peptides known to induce cell detachment in mesothelial cells [201]. Plasma levels of AGE are elevated in patients with chronic renal disease [246] and these may also play a role in mesothelial cell denudation although further studies are warranted to confirm this. In predialysis diabetic patients, it is also possible that increased tissue levels of TGF-*β*1 may contribute to mesothelial cell detachment. Is it therefore possible to obtain adequate quantities of mesothelial cells from uremic patients to store for future transplantation? Given that mesothelial cells in culture have a defined life-span and enter senescence after the second to third passage, is it possible to collect sufficient mesothelial cells that maintain their polygonal morphology without a loss of their proliferative potential? Growth factors may be added to maintain their proliferative capability but this may also increase their fibrogenic potential. Accumulating evidence suggests that mesothelial progenitor cells exist, which may be harvested to assist in the repair and regeneration of the denuded mesothelium [247].

Much of the structural changes that are observed in the peritoneal membrane are induced by the bioincompatible nature of PD fluids. The use of PD solutions with alternative osmotic agents or partial or complete replacement of lactate-buffer with bicarbonate may be more beneficial in preserving the structural and functional integrity of the peritoneal membrane. A recent study demonstrated that PD patients using bicarbonate-buffered, neutral pH PD solutions showed a reduction in the frequency of peritonitis compared to conventional glucose-based, lactate buffered PD fluids [248], but these data have yet to be reproduced.

Although peritonitis exacerbates structural changes to the peritoneal membrane, the initial insult to the peritoneal membrane is the bioincompatible nature of PD fluids resulting in the induction of peritoneal inflammation. In an attempt to halt or even reverse peritoneal injury during PD and peritonitis, is it feasible to rest the peritoneum? Zhe et al. demonstrated that overnight peritoneal rest can improve ultrafiltration capacity in stable PD patients who had been on PD for more than 3 months [249]. In a separate study, Rodrigues et al. observed a recovery in ultrafiltration following peritoneal rest in 8 of 12 PD patients who had developed hyperpermeability [250]. In experimental models of PD, a period of 4–12 weeks of peritoneal resting was associated with a marked reduction in peritoneal fibrosis and angiogenesis, and complete remesothelialization of the peritoneal membrane [251, 252].

## 8. Conclusions

Despite considerable improvement in PD over the past 3 decades, peritonitis remains one of the major complications of PD and is an important cause technique failure and unfavorable clinical outcomes. A frequent cause of peritonitis is contamination at the time of exchange with Gram-positive bacteria that originate from the skin flora. Numerous studies have highlighted the critical role of *Staphylococcus *species in mediating mesothelial cell injury, denudation, and cell death, which leads to increased fibrin and matrix protein accumulation, and ultimately peritoneal fibrosis. Repeated episodes of peritonitis will aggravate these processes and accelerate catheter removal and technique failure. Gram-negative complicating peritonitis is less common that Gram-positive infections but is associated with higher rates of death, hospitalization, and transfer to hemodialysis compared to Gram-positive peritonitis [9]. Our preliminary studies have demonstrated that mesothelial cell denudation is more pronounced when they are exposed to dialysis effluent from PD patients with Gram-negative peritonitis, attributed in part to the higher dialysate levels of proinflammatory cytokines compared to Gram-positive peritonitis [27]. One should therefore bear in mind that different species of microorganisms induce distinct changes to the mesothelium and submesothelium and therefore should not be grouped as one. Furthermore, for experimental and *in vitro* studies simulating PD-related peritonitis, it is noteworthy that subtle structural and regulatory changes to laboratory-based bacteria may result in alterations in their invasiveness and cellular behavior, which are not observed in the clinical setting.

**Figure 1:**
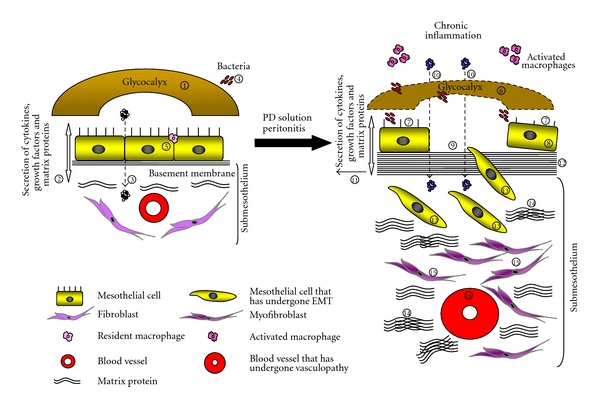
Mesothelial cells line the peritoneal membrane and play a crucial role in peritoneal homeostasis. Their apical surface is endowed with a glycocalyx that provides a protective barrier against abrasion, and a slippery, nonadhesive surface for intracoelomic movement (1). Through their ability to synthesize various cytokines, growth factors, and matrix protein components, mesothelial cells actively participate in tissue repair and induction and resolution of peritoneal inflammation (2). Synthesis of matrix proteins by mesothelial cells may be incorporated into the underlying basement membrane on which mesothelial cells adhere to. Mesothelial cells facilitate in the transport of fluids and solutes across the peritoneal membrane (3), are the first line of defense against bacterial peritonitis (4), and can maintain a chemotactic gradient to assist in leukocyte infiltration (5) during peritoneal inflammation. The submesothelium contains sparse fibroblasts, collagen fibrils and capillaries. Changes to the structural integrity of the peritoneal membrane are invariably observed in PD patients. Constant exposure of the peritoneum to PD fluids, together with peritonitis, results in a reduction of the glycocalyx volume and a concomitant loss of anionic charge in the glycocalyx (6). Alterations in the anionic charge of the peritoneum can result in the reduction in the length and density of microvilli on the surface of mesothelial cells (7). Chronic exposure to PD fluid and peritonitis can induce detachment of mesothelial cells from their underlying basement membrane (8) resulting in partial (9) or complete denudation of the mesothelium. A loss of cell-cell interaction between mesothelial cells permits PD fluid to enter into the submesothelium (10). Increased synthesis of proinflammatory cytokines and matrix proteins is observed following the activation of infiltrating and resident peritoneal cells (11), leading to morphological changes such as reduplication of the basement membrane (12), induction of EMT in mesothelial cells, a breakdown of the basement membrane and their migration into the submesothelium (13). Transdifferentiated mesothelial cells have a greater fibrogenic potential and thus contribute to the deposition of matrix proteins and fibrin in the submesothelium (14), which if not controlled will lead to thickening of the submesothelium and ultimately peritoneal fibrosis and sclerosis. A loss of the protective mesothelium allows PD fluid and toxins released by bacteria to induce the activation of peritoneal fibroblasts (15), hyalinization of blood vessels, and vasculopathy (16). Such detrimental changes to the peritoneal membrane will significantly suppress the dialytic potential of the peritoneal membrane, which will invariably lead to the cessation of treatment.

With a greater understanding of the underlying mechanisms through which different species of microbes can modulate mesothelial cell function and their attachment to the peritoneal membrane during peritonitis, it is envisaged that in time we may devise novel therapeutic interventions to preserve the structural and functional integrity of the peritoneum and thereby improve patient survival on PD.  Figure 1 is a schematic diagram that highlights our current knowledge of how PD and peritonitis may affect the structural integrity of the peritoneal membrane.

## Figures and Tables

**Table 1:** Induction of peritoneal fibrosis.

Phases of peritoneal fibrosis	Events that occur in the peritoneum during each phase
Induction phase	Release of chemokines by mesothelial cells
	(i) Infiltration of mononuclear cells
	(ii) Release of profibrotic mediators
	(iii) Activation of resident cells (mesothelial cells and fibroblasts)
Fibrogenic phase	Increased synthesis and deposition of matrix
	Continued secretion of profibrogenic mediators by infiltrating cells

Peritoneal destruction phase	Cessation of primary inflammatory stimulus
	Secretion of profibrotic cytokines by mesothelial cells
	Autocrine proliferation of fibroblasts and myofibroblasts
	EMT
	Submesothelial thickening
	Vasculopathy
